# Postoperative Nausea and Vomiting After Open Lumbar Discectomy: A Secondary Analysis of a Randomized Trial Using Adequacy of Anesthesia Monitoring

**DOI:** 10.3390/jcm15010360

**Published:** 2026-01-03

**Authors:** Michał J. Stasiowski, Karolina Ćmiel-Smorzyk, Nikola Zmarzły

**Affiliations:** 1 Chair and Department of Emergency Medicine, Faculty of Medical Sciences in Zabrze, Medical University of Silesia, 40-055 Katowice, Poland; 2 Department of Anaesthesiology and Intensive Care, St Barbara 5th Regional Hospital, Trauma Centre, 41-200 Sosnowiec, Poland; 3 Department of Neurosurgery, Medical University of Silesia, Regional Hospital, 41-200 Sosnowiec, Poland; karolina.cmiel-smorzyk@sum.edu.pl; 4 Collegium Medicum, WSB University, 41-300 Dabrowa Gornicza, Poland; nikola.zmarzly@wsb.edu.pl

**Keywords:** open lumbar discectomy, postoperative pain perception, postoperative nausea and vomiting, Adequacy of Anesthesia, infiltration anesthesia, surgical pleth index, bupivacaine, ropivacaine, numeric pain rating scale

## Abstract

**Background/Objectives**: Postoperative nausea and vomiting (PONV) remains a frequent and clinically relevant complication following open lumbar discectomy (OLD) under general anesthesia. The present study represents a secondary, post hoc analysis of a randomized controlled trial originally designed to investigate the effects of infiltration anesthesia (IA) on postoperative pain perception and opioid consumption. The objective of this analysis was to explore the incidence of PONV in patients undergoing OLD under adequacy of anesthesia (AoA)-guided general anesthesia, with or without IA. **Methods**: This secondary analysis included 94 patients undergoing OLD under AoA-guided general anesthesia with fentanyl titration based on the surgical pleth index (SPI). Patients were randomized to receive IA with 0.2% ropivacaine (RF) or bupivacaine (BF) plus 50 µg fentanyl, or no IA (control). PONV was assessed as early (in the post-anesthesia care unit), late (in the neurosurgical ward), and overall (within 48 h postoperatively). Opioid consumption and Apfel risk scores were also analyzed. All analyses related to PONV were exploratory. **Results**: PONV occurred in 12.8% of patients, with no significant differences between study groups. Postoperative morphine consumption was significantly lower in the RF group than in the control group (2.7 ± 5.3 mg vs. 7.1 ± 5.9 mg; *p* < 0.05). Higher pre-induction SPI values were observed in patients who experienced early PONV (73.1 ± 9.7 vs. 59.5 ± 17.2; *p* < 0.05); however, this exploratory finding requires confirmation in larger studies. **Conclusions**: In this secondary, post hoc analysis, no significant differences in PONV incidence were observed between anesthetic groups in patients undergoing OLD under AoA-guided general anesthesia. The observed association between pre-induction SPI values and early PONV should be interpreted cautiously and requires confirmation in adequately powered prospective studies.

## 1. Introduction

Postoperative nausea and vomiting (PONV) remain a common and burdensome complication after surgery under general anesthesia (GA). They impair recovery quality, reduce patient satisfaction [1], prolong hospital stay [2], may lead to unexpected readmissions [3], and increase healthcare costs [4]. Consequently, considerable attention and resources are dedicated to patients at high risk of PONV [5], including both pharmacological prophylaxis [6] and non-pharmacological strategies [7].

The mechanisms underlying PONV are multifactorial [8]. Risk factors have been extensively studied to optimize perioperative care, particularly in patients with a higher predicted risk of PONV [9]. Female gender, non-smoking status, motion sickness, and prior history of PONV, summarized in the Apfel score [10], as well as surgery duration over 60 min, reflected in the Koivuranta score [11], are consistently recognized as major predictors. Additional factors include type of surgery [12], low body mass index [13], volatile anesthetics [14], nitrous oxide in ventilation gas [15], and perioperative use of opioid analgesia [16].

Adequacy of Anesthesia (AoA) is a concept of digital GA monitoring consisting of entropy EEG, surgical pleth index (SPI), and neuromuscular transmission, based on train-of-four stimulation. Entropy EEG incorporates two indices: response entropy (RE; 0–100), calculated from EEG and frontal electromyogram, and state entropy (SE; 0–91), calculated mainly from EEG [17]. Both indices are derived from a frontal sensor applied to the patient’s forehead without complex preparation. The EEG signal is transformed into a digital score in real-time and guides the hypnotic component of GA: values > 80 indicate wakefulness; 60–80, sedation; 40–60, adequate surgical depth; 30–40, excessively deep anesthesia; and < 30, overdose of hypnotic drugs [18]. 

The SPI is a normalized score derived from the heart beat interval (HBInorm) and plethysmographic pulse wave amplitude (PPGAnorm), calculated as SPI  =  100 − [0.7  ×  PPGAnorm  +  0.3  ×  HBInorm]. It ranges from 0 to 100, with higher values indicating intense intraoperative nociception and insufficient antinociception. A return of SPI to baseline after administration of intraoperative rescue opioid analgesia (IROA) reflects adequate analgesia. Thus, SPI monitoring provides real-time assessment of the sympathetic tone of the autonomic nervous system and correlates with the balance between nociceptive stimuli and serum opioid concentration [19,20,21]. SPI is favored in clinical practice because it is derived from standard pulse oximetry, enabling non-invasive, continuous monitoring. Compared to other nociception monitors, SPI has demonstrated greater intraoperative opioid-sparing effects [22] and has been associated with reduced PONV risk [23].

Besides individual risk factors, anesthetic techniques influence PONV incidence. Adverse events following OLD are frequently reported; however, preemptive analgesic modalities such as intravenous [6,10] or regional anesthesia techniques [24], including spinal [25], epidural [26], and infiltrative anesthesia (IA) [27] are commonly used to alleviate moderate-to-severe postoperative pain, decrease IROA requirements, and consequently lower the incidence of PONV, which can reach up to 43.5% of patients under GA [28].

The original randomized trial was designed to investigate the effects of different IA mixtures on postoperative pain perception, perioperative hemodynamic stability, IROA requirements, and postoperative morphine (MF) consumption in patients undergoing OLD under AoA-guided GA as primary outcome measures [29].

The present study is a secondary, post hoc analysis of that trial, focusing specifically on the incidence of PONV, a clinically relevant outcome not included as a primary endpoint. Based on the clinical relevance of PONV and its known association with perioperative opioid exposure and anesthetic management [30,31,32,33], we performed an exploratory analysis to assess the incidence of PONV in patients undergoing OLD under AoA-guided GA, with or without IA.

Additionally, inspired by observations from previous studies using AoA monitoring [32,34,35] and reports suggesting a relationship between preoperative stress and PONV [36], we exploratively examined the association between pre-induction surgical pleth index values and the occurrence of PONV. This analysis was not pre-specified in the original trial protocol and should be regarded as hypothesis-generating.

## 2. Materials and Methods

### 2.1. Patients

Patients scheduled for OLD at the Department of Neurosurgery (DoN) were enrolled between 29 January 2017 and 17 April 2017. 99 of 100 eligible patients (one declined participation) aged 18–80 years, with American Society of Anesthesiologists physical status I–III, were randomized into three groups: (1) IA with 10 mL 0.2% ropivacaine plus 50 μg fentanyl bilaterally at each vertebral segment (Group RF), (2) IA with 10 mL 0.2% bupivacaine plus 50 μg fentanyl bilaterally (Group BF), and (3) IA with 10 mL 0.9% saline solution bilaterally (Group C), similar to the protocol described by Hazarika et al. [37]. Randomization was performed using sealed envelopes by the principal investigator (M.S.) after obtaining written informed consent. The study was conducted in compliance with the Declaration of Helsinki and approved by the Bioethical Committee of the Medical University of Silesia (KNW/0022/KB1/14, 16 December 2014). The study was registered in the Clinical Trials Registry (Silesian MUKOAiIT1, NCT02971540).

Exclusion criteria included pregnancy, allergy to local anesthetics, cardiovascular disease such as arrhythmia or generalized atherosclerosis, and conditions predisposing to intraoperative hypotension, including impaired left ventricular ejection fraction (EF < 40%), mitral or aortic valve stenosis or failure, which could necessitate fluid resuscitation or vasoactive drugs that could interfere with SPI monitoring.

### 2.2. Anesthetic Technique

Before surgery, patients were instructed in the use of the Numeric Pain Rating Scale (NPRS, 0–10) and fasted for 12 h. They were given 3.75–7.5 mg midazolam orally as premedication on the day of surgery. Prior to induction, Optilyte Solution (10 mL/kg; Fresenius Kabi, Warsaw, Poland) was administered intravenously. Following preoxygenation, FNT (2 μg/kg) and propofol (2–2.5 mg/kg) were given intravenously. Mask ventilation was minimized to reduce the risk of PONV. After loss of the ciliary reflex, rocuronium (0.6 mg/kg) was administered and endotracheal intubation performed. Ventilation was adjusted to maintain end-tidal CO_2_ between 35 and 37 mmHg. Sevoflurane was administered with a low-flow technique (0.6 L/min fresh gas: air/oxygen 1:2). The concentration was initially set at 8 vol% and adjusted to achieve minimum alveolar concentration of 0.7–0.8 and a SE of 40–50.

Intraoperative monitoring included non-invasive arterial pressure, heart rate, pulse oximetry, fraction of inspired oxygen, end-tidal CO_2_, electrocardiography, fraction of inspired and expired sevoflurane, minimum alveolar concentration of sevoflurane. AoA was monitored with the Carescape B650 system (GE Healthcare, Helsinki, Finland), using entropy EEG (RE, SE), SPI, and neuromuscular transmission. Rocuronium supplementation was given when the train-of-four count was three or more.

#### 2.2.1. Stage 1

In the operating room, the entropy sensor was applied to the forehead. The SPI pulse oximeter was placed on a finger contralateral to venous access, the noninvasive blood pressure cuff on the right arm, and ECG leads on the patient’s back. Baseline values were recorded.

#### 2.2.2. Stage 2

Following intubation, urinary catheterization was performed and patients were turned to the prone position. SPI values were recorded from 5 min after positioning until skin preparation, and mean values were calculated. IA was performed at the planned incision site according to Hernández-Palazón et al. [38]. The solution was injected into skin, subcutaneous tissues, paraspinal muscles up to the transverse process. Additional injections were made 2 cm posterior to the transverse process into the paravertebral space. IA and discectomy were performed by a consistent team of three neurosurgeons with >10 years of experience. The surgeon adjusted incision placement to the IA site. Both the neurosurgeon performing IA and the anesthesiology team were blinded to the type of IA used. IA solutions were prepared by the principal investigator (M.S.), who was not involved in surgical or anesthetic procedures. The time required for IA was not included in surgical duration.

#### 2.2.3. Stage 3—Intraoperative

Surgical duration was measured from skin incision to final suture. During surgery, SPI was monitored continuously and recorded every minute. If SPI increased by more than 15 points above the Stage 2 mean, a rescue dose of FNT (1 μg/kg) was given intravenously every 5 min until SPI returned to baseline. The initial FNT dose (2 μg/kg) was assumed to provide sufficient analgesia for incision, in addition to preemptive IA in RF and BF groups.

Previous studies suggested ΔSPI > 10 or absolute SPI > 50 as indicators of inadequate analgesia [19,39]. To avoid excessive dosing, we selected ΔSPI > 15 as the threshold for rescue analgesia.

Standard surgical technique included a midline incision (3–5 cm) over the target lumbar segment, discectomy with careful nerve root decompression, and closure with resorbable sutures. Patients were returned supine, neuromuscular blockade was reversed, and extubation was performed. Recovery was defined as an Aldrete score of 9–10 before transfer to the post-anesthesia care unit (PACU).

#### 2.2.4. Stage 4—Postoperatively

In the PACU, monitoring included SPI, heart rate, blood pressure, and oxygen saturation. Supplemental oxygen was provided at 3 L/min and fluid boluses administered as needed to maintain mean arterial pressure (MAP) above 65 mmHg. Pain was assessed every 10 min using the NPRS. If the score exceeded 3, MF 0.03 mg/kg IV [40] was given every 10 min until NPRS fell below 4, according to the guidelines issued by the Polish Society of Anaesthesiologists [41]. SPI was continuously monitored and mean values were recorded every minute. Pain was classified as mild (0–3), moderate (4–6), or acute (7–10). After transfer to the DoN, analgesia was continued according to regulations [41,42].

### 2.3. Apfel Score

Apfel scores were calculated preoperatively to ensure group homogeneity. Risk factors include female gender, non-smoking status, history of motion sickness or PONV, and postoperative opioid administration. Estimated PONV incidences are 10%, 21%, 39%, 61%, and 79% for zero, one, two, three, or four risk factors, respectively [43]. Patients were classified as “low” (0–1 risk factors), “medium” (2), or “high risk” (≥3) [10,44]. The Koivuranta score was not used, as all surgeries exceeded 60 min and were performed with volatile anesthesia and benzodiazepine premedication, which were considered confounders.

PONV was categorized as early (occurring in PACU) or late (occurring in DoN). Overall PONV was defined as any episode within 48 h postoperatively. Intravenous antiemetic treatment was given whenever PONV occurred: dexamethasone 4 mg (Dexaven, Jelfa, Jelenia Góra, Poland) for nausea, and ondansetron 4 mg (Ondansetron Accord, Accord Healthcare Limited, London, UK) in addition to dexamethasone for vomiting. Discharge from PACU to the DoN required four conditions: NPRS < 4 at rest, MAP > 65 mmHg, heart rate 60–90 bpm, and Aldrete score > 8.

### 2.4. Statistical Analysis

The sample size was estimated at 100 based on the total number of surgeries performed (average *n* = 135 per 18 months), a 95% confidence level, a 5% margin of error, and the expected proportions of patients with moderate (NPRS 4–6) and severe pain (NPRS > 6) in a preliminary assessment (26.7% and 43.3%, respectively; *n* = 30).

Statistical analyses were conducted using STATISTICA (ver. 13.3, StatSoft, Kraków, Poland). Continuous variables are presented as mean ± standard deviation and median with interquartile range. Data distribution was assessed with the Shapiro–Wilk test. Comparisons among multiple groups were performed using the Kruskal–Wallis test, followed by Dunn’s post hoc test, while comparisons between two groups employed the Mann–Whitney U test. Categorical variables are presented as percentages and were compared using the Chi-square test, or Fisher’s exact test when expected frequencies were <5. Bonferroni correction was applied for multiple comparisons. A multivariable logistic regression analysis was performed to explore factors independently associated with early PONV. Based on established PONV risk factors and reviewer suggestions, sex, smoking status, and opioid use were included as potential confounders. Pre-induction SPI was included as an exploratory variable. A *p*-value < 0.05 was considered statistically significant.

## 3. Results

A total of 94 patients were included in the study, comprising 42 women (44.7%) and 52 men (55.3%). Patients were allocated into three groups: Control (C, *n* = 31, 33%), Bupivacaine/Fentanyl (BF, *n* = 32, 34%), and Ropivacaine/Fentanyl (RF, *n* = 31, 33%). As reported in our previous study [29], no significant differences were observed among groups regarding age, height, weight, or body mass index (Table S1).

Detailed characteristics of postoperative pain perception and opioid consumption in the study groups are presented in Table 1. The maximum NPRS score was significantly higher in the C group compared to the BF and RF groups, and postoperative MF consumption was greatest in the C group, particularly in comparison with the RF group.

The incidence of PONV is summarized in Table 2. Overall PONV occurred in 12 of 94 patients (12.8%), with no significant differences observed between treatment groups. Early PONV was most frequently observed in patients with intermediate (39%) and high (61%) predicted risk based on the Apfel score. Overall and late PONV were significantly more common in patients with an Apfel score of 3 compared to those with scores of 0 or 1, and early PONV incidence was higher in patients with an Apfel score of 3 compared with a score of 1.

**Figure 1:**
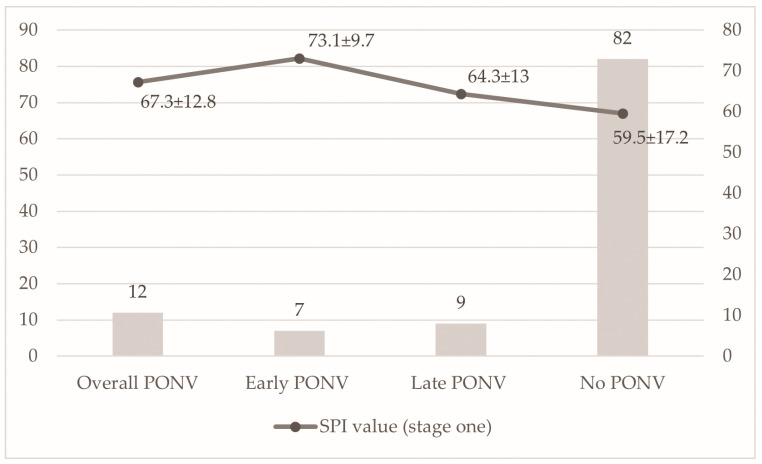
Incidence of PONV categories (overall, early, late, and none) and mean SPI values. The left y-axis represents the number of patients (*n*), and the right y-axis represents mean SPI values (±SD).

In an exploratory univariable analysis, patients who developed early PONV had higher pre-induction SPI values (Stage 1) than those without early PONV (73.1 ± 9.7 vs. 59.5 ± 17.2) (Figure 1). However, in multivariable logistic regression analysis adjusted for sex, history of motion sickness, smoking status, and pre-induction SPI, none of the included variables were independently associated with early PONV.

## 4. Discussion

Reducing the incidence of PONV in patients undergoing various surgical procedures remains a longstanding challenge, including in patients undergoing OLD under GA [45]. PONV impairs patient satisfaction and motivates the implementation of multiple anesthetic strategies to improve perioperative outcomes [46,47,48].

The employment of enhanced recovery after surgery, a multimodal approach aimed at optimizing recovery after single-level lumbar microdiscectomy, has been shown to reduce PONV incidence [9]. Blumenthal et al. demonstrated that improved intraoperative pain control using controlled-release oxycodone during elective OLD over 1–2 levels significantly reduced PONV during the first 24 h compared to placebo [49]. Similarly, Aveline et al. compared three preemptive analgesic modalities, including intravenous MF, ketamine, or a combination of MF with ketamine. Postoperative pain scores were lower and PONV incidence decreased from 43.5% to 21.7% (*p* = 0.001) in the ketamine/MF group due to better pain control and reduced postoperative opioid demand [28].

However, reports on the effectiveness of different preemptive analgesic techniques in reducing PONV after OLD remain inconsistent [50]. Some studies have reported lower PONV incidence in patients receiving IA with bupivacaine, alone or with adjuvants [51], or combined GA-epidural [52]. Local anesthetics exert their effects through reversible sodium channel blockade, thereby inhibiting nociceptive signal transmission. Ropivacaine, a long-acting amide local anesthetic, selectively targets nociceptive fibers and is associated with a more favorable safety profile compared with bupivacaine [53]. Preoperative IA at the concentrations used (0.2–0.25%) provided effective selective sensory block for postoperative analgesia but was insufficient to suppress intraoperative nociception without rescue opioids [54,55,56]. Alternative regional anesthesia techniques, such as spinal anesthesia or interfascial plane blocks, have been reported to reduce PONV compared to GA in high-risk patients [26,57,58,59,60,61]. Similarly, goal-directed opioid titration using analgesia/nociception indices has been associated with lower postoperative nausea scores [62].

In the present secondary, post hoc analysis, the overall PONV incidence was observed in 12 of 94 patients (12.8%), which is substantially lower than the approximately 40% incidence reported in other studies following OLD. Importantly, no statistically or clinically meaningful differences in overall, early, or late PONV incidence were observed between the studied groups, despite significant differences in postoperative pain intensity and morphine consumption. These findings suggest that, within the limitations of this analysis, the type of IA used, or its absence, did not independently influence PONV occurrence when anesthesia was conducted under standardized AoA guidance.

As expected, PONV incidence increased with higher Apfel scores, confirming the validity of established PONV risk stratification in this cohort. This observation aligns with extensive prior literature [30,31,32,63,64] and does not represent a novel finding, but rather reinforces the multifactorial nature of PONV and the continued relevance of baseline patient-related risk factors.

Taken together, available evidence suggests that adequacy-of-anesthesia-guided techniques, including SPI- and entropy-based monitoring, primarily contribute to improved titration of hypnotic and opioid agents rather than exerting a direct, independent effect on PONV incidence. Previous studies have demonstrated that SPI-guided analgesia may reduce intraoperative opioid consumption and improve postoperative pain control [23,34], while entropy EEG monitoring ensures adequate hypnotic depth and may reduce anesthesia-related adverse events [65,66]. However, data regarding a consistent and clinically meaningful impact of these monitoring strategies on PONV remain heterogeneous across studies, with some studies reporting lower nausea scores [23] and others failing to confirm a significant benefit [67,68]. In this context, the low overall PONV incidence observed in the present study likely reflects standardized AoA-guided anesthetic management rather than the effect of a specific analgesic technique or monitoring parameter.

An exploratory observation of this analysis was the higher pre-induction SPI values in patients who developed early PONV. However, this association was not sustained after adjustment for established risk factors in multivariable logistic regression analysis. Given the post hoc nature of the analysis and the limited number of PONV events, this finding should be interpreted cautiously and regarded as hypothesis-generating only. While SPI values have been explored as markers of nociception–antinociception balance at various perioperative stages, evidence regarding their impact on PONV remains inconsistent, and the present data do not support SPI as an independent prognostic marker of PONV.

Future research could focus on integrating digital anesthesia monitoring, including SPI and entropy EEG [34,69,70], to further explore potential associations with PONV risk. Non-pharmacological interventions aimed at reducing preoperative anxiety and sympathetic activation, such as hypnosis [71], acupuncture [72,73], acupressure [74], or meditation [75], may serve as complementary strategies in selected patients. Further studies in larger patient cohorts are needed to validate these approaches and establish evidence-based, individualized strategies for PONV prevention.

Several limitations of the present analysis must be acknowledged. First, the study was not originally designed or powered to assess differences in PONV incidence, and the number of observed events was small, limiting the robustness of statistical analyses. Second, SPI monitoring was applied uniformly to all patients as part of the anesthesia protocol and was not a randomized intervention; therefore, any associations between SPI values and PONV could only be explored observationally. Third, all patients received sevoflurane, a known risk factor for PONV, which may have influenced the overall incidence [76]. Fourth, late PONV may have been underreported due to the subjective nature of symptoms. Fifth, perioperative factors such as oral midazolam premedication and liberal fluid management may have contributed to the relatively low PONV rates observed. Finally, the threshold of ΔSPI > 15 for rescue FNT administration, although chosen to avoid overdosing, differs from alternative cut-offs used in other studies (ΔSPI > 10 or absolute SPI > 50), which may limit direct comparability of results [19].

## 5. Conclusions

In this secondary analysis, no significant differences in PONV incidence were observed between anesthetic groups in patients undergoing open lumbar discectomy under AoA-guided general anesthesia. Higher Apfel scores were associated with increased PONV incidence, consistent with the established literature. Exploratory observations regarding pre-induction SPI values were not confirmed in multivariable analysis and should be interpreted as hypothesis-generating. Further prospective studies are needed to determine whether AoA-guided anesthesia may indirectly contribute to PONV reduction and to clarify the potential role of autonomic markers in perioperative risk stratification.

## Figures and Tables

**Table 1:** Apfel scores and patient history by group allocation.

Scale		Total	C Group	BF Group	RF Group	*p*-Value
		*N* = 94 (100%)	*n* = 31 (33%)	*n* = 32 (34%)	*n* = 31 (33%)	
Apfel X ± Sd Me (IQR)	pts	1.39 ± 0.86 1(1)	1.52 ± 0.89 1(1)	1.34 ± 0.83 1(1)	1.32 ± 0.87 1(1)	0.7 NS
	%	29.48 ± 14.86 21(18)	31.6 ± 16.1 21(18)	28.5 ± 13.8 21(18)	28.3 ± 14.9 21(18)	0.7 NS
NPRS X ± Sd Me (IQR)	min	1.9 ± 1.57 2(3)	2.48 ± 1.77 2(1)	1.44 ± 1.41 2(3)	1.81 ± 1.38 2(3)	0.1 NS
	max	4.17 ± 3.08 3.5(5)	5.55 ± 3.04 6(5)	3.72 ± 3.26 3.5(7)	3.26 ± 2.48 3(2)	C vs. BF, *p* = 0.047; C vs. RF, *p* = 0.01
Moderate-to-severe pain NPRS > 3 *n* (%)	no	48 (51.07%)	10 (32.3%)	17 (53.1%)	21 (67.7%)	C vs. RF, *p* = 0.03
	yes	46 (48.93%)	21 (67.7%)	15 (46.9%)	10 (32.3%)	
Motion sickness *n* (%)	no	93 (98.9%)	31 (100%)	31 (96.9%)	31 (100%)	-
	yes	1 (1.1%)	0 (0%)	1 (3.1%)	0 (0%)	
Smoking *n* (%)	no	51 (54.3%)	16 (51.6%)	14 (43.8%)	21 (67.7%)	0.2 NS
	yes	43 (45.7%)	15 (48.4%)	18 (56.2%)	10 (32.3%)	
Postoperative use of opioid drugs *n* (%)	no	55 (58.5%)	12 (38.7%)	19 (59.4%)	24 (77.4%)	C vs. RF, *p* = 0.005
	yes	39 (41.5%)	19 (61.3%)	13 (40.6%)	7 (22.6%)	
Postoperative MF consumption in the PACU X ± Sd Me (IQR)	mg	4.6 ± 5.66 0(10)	7.1 ± 5.93 8(12)	4 ± 4.93 0(10)	2.71 ± 5.33 0(0)	C vs. RF, *p* = 0.009
Intraoperative FNT requirement X ± Sd Me (IQR)	µg	511.17 ± 388 400(450)	491.94 ± 244.64 500(400)	623.44 ± 548.01 500(525)	414.52 ± 270.24 350(400)	0.2 NS

C—control group, BF—bupivacaine/fentanyl group, RF—ropivacaine/fentanyl group, NPRS—numerical pain rating scale, PACU—post-anesthesia care unit, MF—morphine, FNT—fentanyl, Sd—standard deviation, Me—median, IQR—interquartile range, NS—not significant.

**Table 2:** Incidence of PONV in patients according to treatment group and Apfel score category.

**Intraoperative Data**			**Total**	**C Group**	**BF Group**	**RF Group**	***p*** **-Value**
			***N* = 94 (100%)**	***n* = 31 (33%)**	***n* = 32 (34%)**	***n* = 31 (33%)**	
Overall PONV *n* (%)		yes	12 (12.8%)	3 (9.7%)	5 (15.6%)	4 (12.9%)	0.9 NS
Early PONV *n* (%)		yes	7 (7.4%)	2 (6.5%)	1 (3.1%)	4 (12.9%)	0.3 NS
Late PONV *n* (%)		yes	9 (9.6%)	2 (6.5%)	5 (15.6%)	2 (6.5%)	0.5 NS
Both early and late PONV *n* (%)		yes	4 (4.6%)	1 (3.2%)	1 (3.1%)	2 6.5%)	0.8 NS
**Apfel Score [Point]** ***n* (%)**	**Low risk of PONV**			**Medium risk of PONV**	**High risk of PONV**	***p*** **-Value**	**First monitored SPI value** **(stage one)**
	**0** **(10% Risk of PONV)** ***n* = 13**		**1** **(21% Risk of PONV)** ***n* = 41**	**2** **(39% Risk of PONV)** ***n* = 30**	**3** **(61% Risk of PONV)** ***n* = 10**		
Overall PONV *n* = 12	0 (0%)		2 (4.9%)	5 (16.7%)	5 (50%)	0 vs. 3, *p* = 0.045; 1 vs. 3, *p* = 0.01	67.3 ± 12.8 70(18.5)
No PONV *n* = 82	13 (100%)		39 (95.1%)	25 (83.3%)	5 (50%)		59.5 ± 17.2 62(29)
Early PONV *n* = 7	0 (0%)		1 (14.3%)	3 (42.9%)	3 (42.9%)	1 vs. 3, *p* = 0.02	73.1 ± 9.7 74(20)
Late PONV *n* = 9	0 (0%)		1 (11.1%)	4 (44.4%)	4 (44.4%)	0 vs. 3, *p* = 0.03; 1 vs. 3, *p* = 0.004	64.3 ± 13 62(18)
*p*-value	-		-	-	-	-	Early PONV vs. No PONV, *p* = 0.03

C—control group, BF—bupivacaine/fentanyl group, RF—ropivacaine/fentanyl group, PONV—postoperative nausea and vomiting, SPI—surgical pleth index, Sd—standard deviation, Me—median, IQR—interquartile range, NS—not significant.

## Data Availability

Data is contained within the article or Supplementary Material.

## Supplementary Materials

The following supporting information can be downloaded at: https://www.mdpi.com/article/10.3390/jcm15010360/s1, Table S1: Anthropometric characteristics of patients in each study group.

## Abbreviations

The following abbreviations are used in this manuscript:

AoA	Adequacy of Anesthesia
BF	Bupivacaine/fentanyl
C	Control
DoN	Department of Neurosurgery
FNT	Fentanyl
GA	General anesthesia
IA	Infiltration anesthesia
IPPP	Inappropriate postoperative pain perception
IROA	Intraoperative rescue opioid analgesics
MAP	Mean arterial pressure
MF	Morphine
NPRS	Numeric pain rating scale
OLD	Open lumbar discectomy
PACU	Post-anesthesia care unit
PONV	Postoperative nausea and vomiting
RE	Response entropy
RF	Ropivacaine/fentanyl
SE	State entropy
SPI	Surgical pleth index
