# Supplementary material for: Postoperative Nausea and Vomiting After Open Lumbar Discectomy: A Secondary Analysis of a Randomized Trial Using Adequacy of Anesthesia Monitoring
Source: J Clin Med. 2026 Jan 3;15(1):360. doi: 10.3390/jcm15010360 (PMC12786424; doi:10.3390/jcm15010360)
Supplement: Supplementary file 1 [file jcm-15-00360-s001.zip › Table S1.pdf]

Journal of Clinical Medicine

Table S1. Anthropometric characteristics of patients in each study group.

Anthropometric data		Total	C Group	BF Group	RF Group	*p*-value
		N **= 94 (100%)**	***n*** **= 31 (33%)**	***n*** **= 32 (34%)**	***n*** **= 31 (33%)**	
Age<br>X±Sd<br>Me (IQR)	years	50.71±13.75<br>51(23)	48.1±12.84<br>46(23)	52.22±13.8<br>52.5(21)	51.77±14.63<br>51(24)	0.53<br>NS
Height<br>X±Sd<br>Me (IQR)	cm	168.82±13.2<br>170(12)	169.61±8.53<br>170(12)	167.22±18.77<br>169.5(12)	169.68±9.97<br>170(11)	0.93<br>NS
Weight<br>X±Sd<br>Me (IQR)	kg	79.75±14.04<br>78.5(22)	80.47±16.75<br>80(30)	80.06±11.22<br>78.5(14)	78.71±14.1<br>78(23)	0.91<br>NS
BMI<br>X±Sd<br>Me (IQR)	kg/m^2	28.86±13.34<br>27.76(5.21)	27.71±4.32<br>27.8(4.66)	31.48±22.19<br>27.64(4.6)	27.36±4.37<br>28.06(7.28)	1.0<br>NS
Gender<br>*N* (%)	Female	42 (44.7%)	12 (38.7%)	16 (50%)	14 (45.2%)	0.66
	Male	52 (55.3%)	19 (61.3%)	16 (50%)	17 (54.8%)	

C – control group, BF – bupivacaine/fentanyl group, RF – ropivacaine/fentanyl group, Sd – standard deviation, Me – median, IQR – interquartile range, BMI – body mass index, NS – not significant.
